# Supplementary figures and images for: A quantitative pipeline to assess secretion of human leptin coding variants reveals mechanisms underlying leptin deficiencies
Source: J Biol Chem. 2024 Jul 19;300(8):107562. doi: 10.1016/j.jbc.2024.107562 (PMC11366920; doi:10.1016/j.jbc.2024.107562)

Supplemental Figure 1

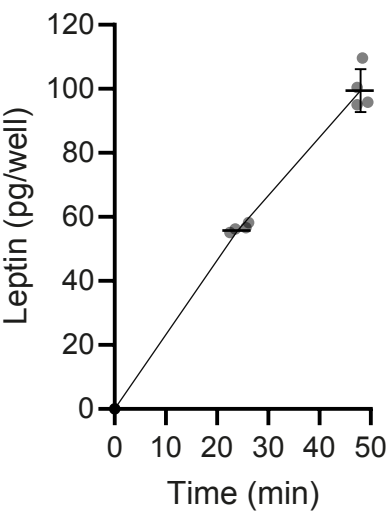

Supplement: Figure S1 [file mmc1.pdf]

Supplemental Figure 2

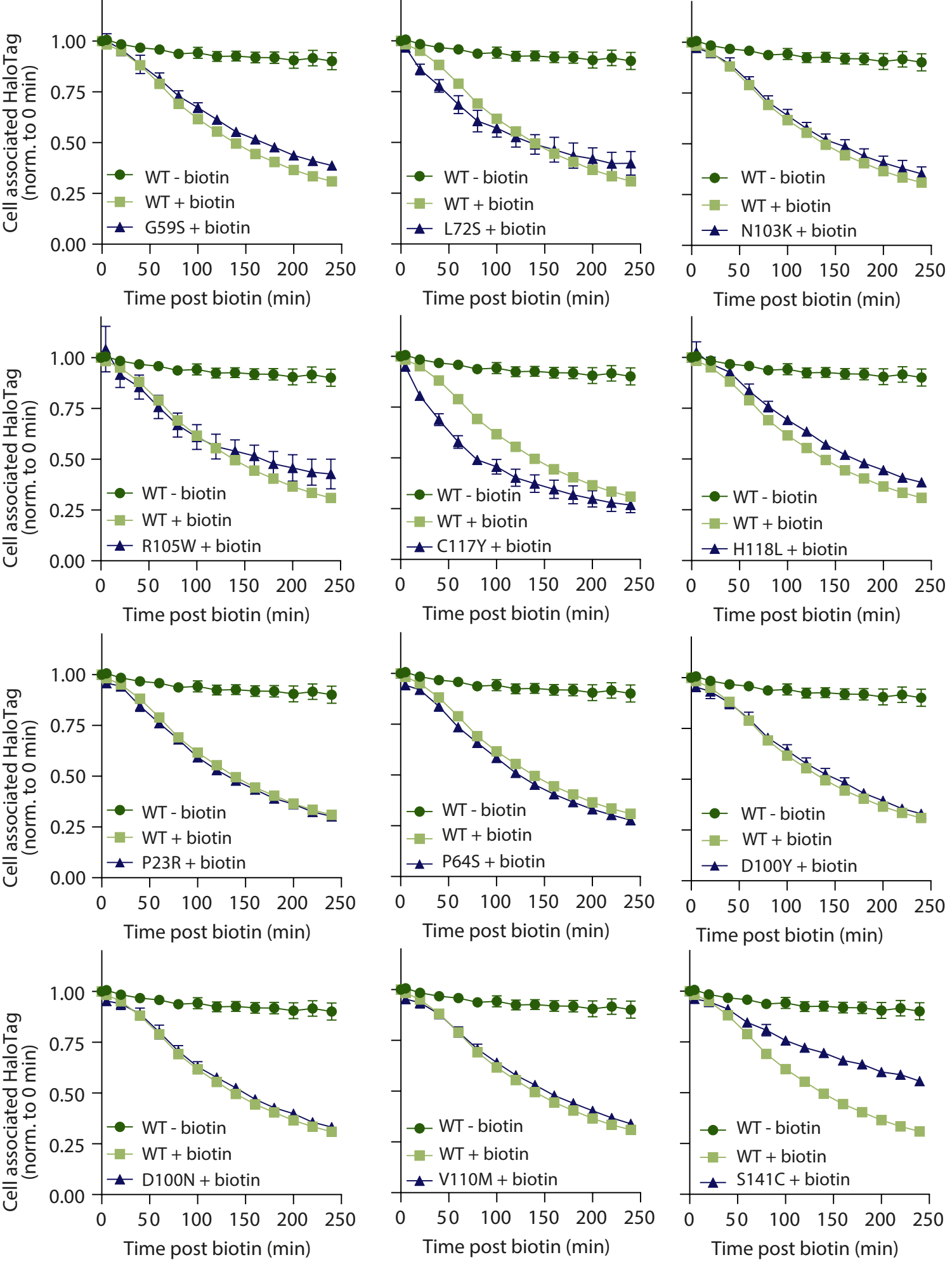

Supplement: Figure S2 [file mmc2.pdf]

Supplemental Figure 3

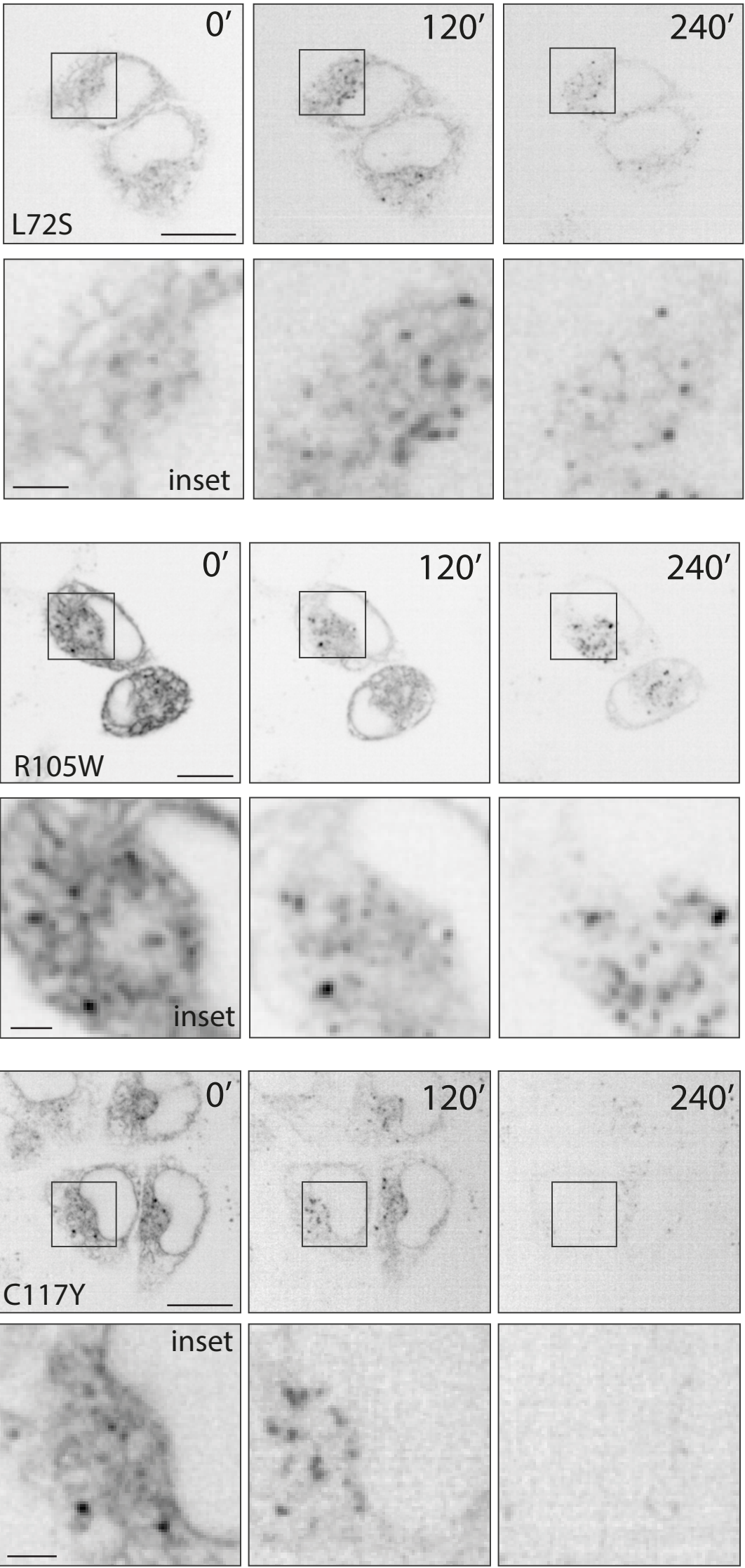

Supplement: Figure S3 [file mmc3.pdf]
