# Supplementary material for: A quantitative pipeline to assess secretion of human leptin coding variants reveals mechanisms underlying leptin deficiencies
Source: J Biol Chem. 2024 Jul 19;300(8):107562. doi: 10.1016/j.jbc.2024.107562 (PMC11366920; doi:10.1016/j.jbc.2024.107562)
Supplement: Supplemental figure legends [file mmc4.docx]

**Supplementary figure 1.** Extracellular endogenous leptin concentrations from 3T3-L1 adipocyte cultures in 12 well plates for the respective time points. 0 h = naive media. n = 4. Error bars = S.E.M.

**Supplementary figure 2**. Leptin variant RUSH-secretion assay screen. Kinetic secretory analysis of HeLa cells expressing WT or the indicated leptin variant. The same WT data is plotted on each graph for ease of comparison. Biotin was added where indicated. Cells were imaged at 20 min time intervals from 0-240 min. Cell-associated HaloTag fluorescence intensity was normalised to the 0 min time point. n= 7 for WT SBP-HaloTag-leptin; n = 3 for all leptin variants. Error bars = S.E.M.

**Supplementary figure 3**. Representative fluorescence micrographs of HeLa cells expressing the SBP-HaloTag-leptin variants indicated before biotin addition and after biotin at the indicated time points. These images were from the datasets quantitatively analysed in Figure S2. Scale bars = 10 µm; inset scale bars = 2 µm.
